# ETS and Conception: Smoking Out a Mechanism of Action

**DOI:** 10.1289/ehp.113-a254a

**Published:** 2005-04-01

**Authors:** Laura Alderson

Numerous studies have shown that women who smoke have a harder time getting pregnant and a greater risk of spontaneous abortion and having low-birth-weight babies. Smoking also has long been associated with menstrual disturbances and possibly other antiestrogen effects. But findings on reproductive outcomes have been less straightforward when it comes to the harmful effects of women’s exposure to environmental tobacco smoke (ETS). Now a team of U.S. and Chinese researchers report a link between ETS exposure and significantly lower urinary levels of estrone conjugates (E_1_C; the main metabolite of estrogen)**[** ***EHP*** **113:412–417]**. The findings suggest that ETS exposure, like active smoking, may affect reproduction in part through antiestrogen effects.

The study was part of a large, prospective reproductive health study conducted from 1997 to 2000 among women working full-time at the Anqing Textile Mill in southeast China’s Anhui Province. In an earlier study of the same cohort, the investigators reported a dose–response relationship between ETS exposure and risk of early pregnancy loss.

This study included 371 newly married women aged 20–34 who had stopped contraception and intended to conceive. None had borne children. All were nonsmoking, all had the same occupation, and all were of the same socioeconomic background. ETS exposure in this homogeneous group was very high because of the high prevalence of smoking among Chinese men. In China, it is estimated that 63% of men are smokers (compared to only 3.8% of women).

For up to a year, or until pregnancy, the women kept daily diaries recording exposure to ETS at both home and work. They also collected first-morning urine specimens each day. The researchers calculated the percentage of days in each menstrual cycle that included ETS exposure, then analyzed the independent association between ETS exposure and profiles of pregnanediol-3-glucuronide (PdG) and E_1_C in the women’s menstrual cycles. PdG and E_1_C are urinary metabolites of hormones that regulate reproductive function.

Because individual women’s hormone levels vary significantly during their menstrual cycles, it was critical to accurately determine the day of ovulation in order to compare hormone levels among the study subjects. The researchers used the estimated day of ovulation to align the individual menstrual cycles for comparison.

Of the 673 cycles included in the study, 344 (51%) were non-conceptive and 329 (49%) resulted in conception. Only 76 cycles did not include ETS exposure; of these, 42% resulted in conception. During nonconception cycles, ETS-exposed women had a consistently lower daily urinary E_1_C level compared to nonexposed women. During conception cycles, the association was not significant; the investigators write that their study may not have had the power to detect a relatively small effect of ETS exposure in these cycles. They also reported no significant difference in PdG levels between women who were exposed to ETS and those who weren’t, regardless of conception status.

These findings suggest that ETS exposure, like active smoking, may affect estrogen levels. Estrogen affects the timing of ovulation during the menstrual cycle, thus potentially affecting the ability to become pregnant. In shedding new light on the biological mechanisms by which ETS exposure may affect reproduction, the study adds to the body of knowledge on the harmful impact of smoking upon smokers and nonsmokers alike.

## Figures and Tables

**Figure:**
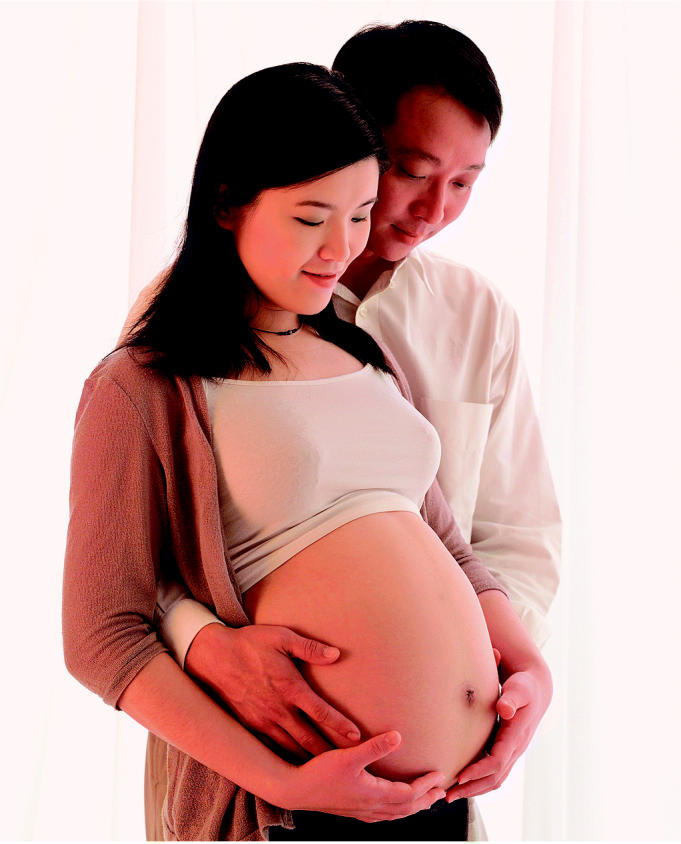
**Conception smokescreen.** A study of Chinese women trying to conceive adds to the body of evidence that ETS exposure—not just active smoking—may hamper reproduction.

